# Open subpectoral biceps tenodesis in patients over 65 does not result in an increased rate of complications

**DOI:** 10.1186/s12891-017-1780-1

**Published:** 2017-11-06

**Authors:** Andreas Voss, Simone Cerciello, Jessica DiVenere, Olga Solovyova, Felix Dyrna, John Apostolakos, David Lam, Mark P. Cote, Knut Beitzel, Augustus D. Mazzocca

**Affiliations:** 1 Department of Orthopaedic Surgery, University of Connecticut Health Center, Farmington, CT USA; 2 Department of Orthopaedic Sports Medicine, Technical University of Munich, Munich, Germany; 3 Department of Orthopaedic Surgery, Casa di Cura Villa Betania, Rome, Italy; 4 Department of Orthopaedic Surgery, Marrelli Hospital, Crotone, Italy; 5 Department of Orthopaedic Surgery, NYU Hospital for Joint Disesases, New York, NY USA

**Keywords:** Shoulder, Subpectoral, Biceps, Tenodesis, Open tenodesis, Over 65

## Abstract

**Background:**

Long head biceps tendon pathology is a common cause of anterior shoulder pain and is often associated with other shoulder conditions, such as rotator cuff tears and osteoarthritis. It is well accepted that older patients are at increased risk for major and minor peri- and postoperative complications.

The purpose of this study is to investigate patients over 65 years old who underwent subpectoral biceps tenodesis and compare the complication rates of this group to those of patients younger than 65 years old. The hypothesis is, that there would be no difference in complication rates and that clinical outcome scores for patients over 65 were satisfying and showed improvements over time.

**Methods:**

There were 337 patients who underwent open subpectoral biceps tenodesis, between January 2005 and June 2015, 23 were identified as being over the age of 65 with a minimum follow up of 12 months. All patients over the age of 65 were evaluated pre- and postoperatively using Simple Shoulder Test (SST), American Shoulder and Elbow Surgeons (ASES), Constant-Murley (CM) and Single Assessment Numeric Evaluation (SANE). Intraoperative and postoperative adverse events (fracture, infection, wound opening, rupture/failure and neurovascular injuries) related to the tenodesis procedure and to the surgery itself were collected from all 337 patients in a routine postoperative follow-up.

**Results:**

The under 65 group (range 27–64 years) at an average follow up (FU) of 30 months (range 12–91 months) showed a 5.4% (17 out of 314) post-operative complication rate related to the subpectoral tenodesis, whereas the group over 65 (range 65–77 years) at an average follow up of 33 months (range 12–79 months) showed an 8.7% (2 out of 23) complication rate.

**Conclusion:**

This study demonstrates that in patients over the age of 65, biceps tenodesis is a successful procedure when performed for biceps tendinopathy and concomitantly with other surgical procedures of the shoulder, and does not result in an increased rate of complications when compared to a group of patients under the age of 65.

## Background

Long head biceps tendon (LHB) pathology is a common cause of anterior shoulder pain and is often associated with other shoulder conditions [1–4]. Therefore, biceps tenodesis is a common and well accepted procedure. The main purpose is to restore the physiological shape of the upper limp and to avoid postoperative cramping of the biceps muscle, as a known symptom after tenotomy. According to Giphart et al. [5] there is no significant difference in motion after a tenotomy compared to intact biceps tendon.

It is well accepted that patients over the age of 65 are at increased risk for major and minor peri- and postoperative complications [6–11]. Although there are no studies that correlate the rate of complications in biceps tenodesis to age, based on the above mentioned data, it seems reasonable to infer that this procedure may have a greater rate of complications with increasing age as well. Risks of fracture during drilling and insertion of the interference screw, wound complications, and venous thromboembolic disease are of particular concern.

Though the evidence is mixed a greater incidence of wound infections in older patients has been described in the literature [12–15], as well as several case reports describing proximal humerus fracture during subpectoral biceps tenodesis [16, 17]. Due to the concern regarding increased rates of complications in older patients, some surgeons elect to perform only biceps tenotomies in these patients. The limited evidence in the literature, reports comparable outcomes for biceps tenodesis versus tenotomy [18–23], though studies show that patients treated with tenotomies have greater incidence of postoperative cramping and cosmetic deformity [18–21, 23].

To our knowledge, there is no published literature evaluating the complications and outcomes of biceps tenodesis in patients older than 65. Therefore, the purpose of this study was to retrospectively evaluate prospectively collected clinical outcomes data in patients over 65 years old who underwent subpectoral biceps tenodesis, to report their clinical outcome data and to compare the complication rates to those of patients younger than 65 years old who had the same procedure performed. Our hypothesis is that there would be no difference in adverse events among patients over 65. Furthermore, we hypothesized, that clinical outcome scores were satisfying and showed improvements over time.

## Methods

This is a retrospective case series of prospectively collected data of all patients 65 or older who underwent open subpectoral biceps tenodesis with an interference screw fixation, between 2005 and June 2015, in a singles surgeon’s practice (*n* = 380). Patients were identified through an outcome registry query (IRB# IE-13-151-1). Patients undergoing concomitant arthroplasty, resurfacing procedure, or revision procedures were excluded (n.43).

All patients were included in the decision process of the surgical procedure regarding tenotomy vs. tenodesis and the patient made the final decision. Indication for tenodesis in patients over 65 years old include: Chronic atrophic changes in the LHBT, painful and therapy resistant tenosynovitis, symptomatic intra-articular partial tears (>25%) of the LHBT, additional treatment during rotator cuff repair surgery, pulley lesion with biceps instability (subluxation and luxation), SLAP lesion in elderly patients, painful and hyperthrophic LHBT with secondary impingement and subpectoral biceps pain. Contraindications for subpectoral biceps tenodesis were: obesity, diabetes, highly osteoporotic bone, increased cardio vascular morbidity, tumor at the proximal humerus and patient with implants (e.g.: plates and nails). Obesity was defined according to the WHO (BMI ≥ 30). Patients who presented with documented back pain, caused by a fractured or collapsed vertebra, loss of height over time, a bone fracture from standing height or a diagnosed osteoporosis through bone mineral density measurements were not eligible for subpectoral biceps tenodesis.

Outcome measures including the Simple Shoulder Test (SST), American Shoulder and Elbow Surgeons (ASES), Constant-Murley (CM) and Single Assessment Numeric Evaluation (SANE) were prospectively collected preoperatively and postoperatively in all patients over the age of 65, including adverse events and postoperative complications. All patients below 65 have been seen and evaluated on a regular basis to determine if any adverse event or postoperative complications occurred, but complete postoperative outcome data (SST, ASES, CM and SANE) was not obtained for all patients. Adverse events including death, venous thromboembolic disease, intraoperative and/or postoperative fracture, intraoperative nerve or vessel damage, superficial and deep surgical site infections, wound dehiscence, repair failure and large postoperative hematoma. Additional information abstracted from the medical record included indications for primary procedure, e.g. persistent pain and shoulder stiffness.

### Surgical technique [24]

After arthroscopic tenotomy of the LHBT, the skin incision is followed by a safe blunt dissection of the pectoralis major tendon until the bicipital groove and the long head of the biceps tendon are exposed. The LHBT is then stitched starting 2 cm from the musculotendinous junction for 2 cm. A guide pin is used to drill a unicortical hole in the ventral aspect of the cortex within the bicipital groove, followed by an 8-mm unicortical reamer. After unicortical drilling an 8-mm tap is used to prepare the cortex. One of the stiches end is then loaded through the biceps tenodesis screwdriver, the other end is left free. An 8-mm screw is deployed along with the tendon into the previously drilled 8 mm hole till the screw is flush with the humeral cortex. The two ends of the suture are then tied over the screw securing the screw in place.

### Statistical analysis

Descriptive statistics to characterize the study group were calculated using means and standard deviation or frequency and proportion where appropriate. No power analysis has been performed because this study is a sample of convenience. Difference between the pre- and postoperative outcome scores in patients ≥65 years of age were compared with a paired *t* test. Rates of adverse events were compared between patients ≥65 and <65 years of age with Fischer’s exact test. The alpha level for all comparisons was set at 0.05 using Stata 12 (StataCorp. 2011. Stata Statistical Software: Release 12. College Station, TX: StataCorp LP).

### Results

**Fig. 1:**
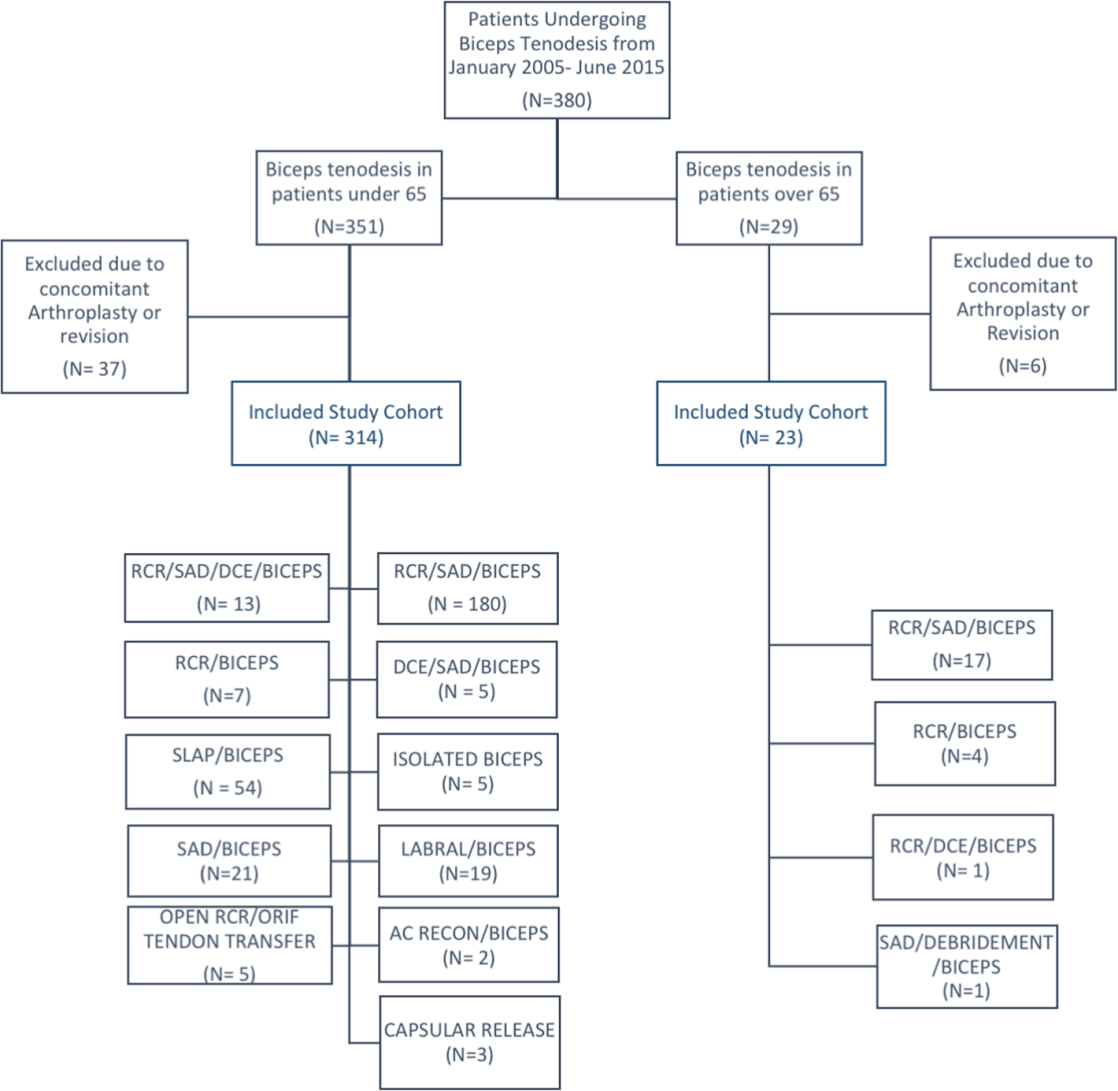
Flowchart showing inclusion and exclusion of the study population

This search resulted in 337 patients of whom 314 patients were included in the under 65 years group and 23 in the over 65 group. The study group consisted of 23 patients (Table 1). The average age at time of surgery was 69.7 years (range 65–77) and the average length of follow-up was 33 months (range 12–79). Biceps tenodesis was associated with a concomitant procedure in all cases (Fig. 1). Two patients (8.7%) had biceps related complications including one biceps tendonitis and one LHB rupture. Three patients (13%) had postoperative complications not strictly related to the tenodesis itself. One developed a wound infection related to the rotator cuff repair, which required arthroscopic irrigation and debridement and antibiotics; the infection resolved, and the patient went on to have no further sequelae and excellent outcomes. Two patients developed a postoperative adhesive capsulitis, related to rotator cuff repair, which resolved with appropriate physical therapy (Table 2). There were no incidences of death, intraoperative fracture, intraoperative nerve or vessel damage, repair failure, or persistent pain.

**Table 1:** Descriptive data of included study population with additional clinical scores for patients over the age of 65

	Over 65 yrs	Under 65 yrs	*P* value
Number of patients	23	314	
Average age	69.7	50	
Average lenght of FU (months)	33	30	
% of complications related to the tenodesis itself	8.3%	5.4%	ns
% of complications not related to the tenodesis itself	12.5%	31.2%	
Increase in ASES score	45.6	na	*p* < 0.001
Increase in Constant score	52.2	na	*p* < 0.001
Increase in SST	5.0	na	*p* < 0.001

In the under 65 cohort (*n* = 314) the average age at the time of surgery was 50 years (range 29–64) and the average FU was 30 months (range 12–91 months). Biceps tenodesis was performed as an isolated procedure in 5 cases or in association with other procedures in 309 cases (see flow-chart diagram). Seventeen patients (5.4%) had complications related to the tenodesis itself (hematoma, granuloma, infection, rupture, pain over the tenodesis). Thirty-eight patients (12.1%) had persistent pain (variable location), 48 (15.3%) had complications related to the cuff repair (adhesive capsulitis, weakness, failure), and 12 (3.8%) had various complications (tingling, post traumatic fracture) (Tab. 2). There were no incidences of death, intraoperative fracture, or intraoperative nerve or vessel damage. The difference in complication rates between the under and over 65 years groups was not statistically significant (*p* = 0.23).

**Table 2:** Overview of complications related to the subpectoral tenodesis in both over and under 65 years

	Over 65 yrs			Under 65 yrs		
Patients	23			314		
Complications related to the tenodesis	n.2 (8.7%)	LHB tendonitis	n.1 (4.3%)	n.17 (5.4%)	hematoma, granuloma, infection, rupture, pain over the tenodesis	n.17 (5.4%)
		LHB rupture	n.1 (4.3%)			
Complications not related to the tenodesis	n.3 (13%)	Adhesive capsulitis	n.2 (8.7%)	n.98 (31.2%)	Persistent pain	n.38 (12.1%)
		Wound infection	n.1 (4.3%)		adhesive capsulitis, weakness, failure	n.48 (15.3%)
					tingling, post traumatic fracture	n.12 (3.8%)

Pre- and postoperative outcomes in the older than 65 group were assessed. The mean pre- and postoperative ASES scores were 45.1 (±19.9) and 90.8 (±16.2), respectively. The SST score increased from 5.6 (±3.1) to 10.6 (±2.2), the CM increased from 37.0 (±13.8) to 89.2 (±9.0) and the post-operative SANE score showed good results with a mean of 89.6 (±15.3). All of these improvements were statistically significant (*p* < 0.001).

## Discussion

The most important finding of the present study is the comparable specific complication rate of subpectoral tenodesis in patients older (8.7%) and younger (5.4%) than 65 years. The general rate of complications was 21.7% in the over 65 years cohort and 36.6% in the under 65 years group. Moreover, the functional outcomes are encouraging with significant improvement in all examined tools (ASES, SST, CM and SANE). The outcomes are comparable to those reported in recent studies on younger patients with persistent pain having been reported in up to 50% of patients. [25, 26].

These findings are particularly interesting since they may help change the management of biceps pathology in this older cohort of patients. In fact, it is well established that pathology of the LHB either traumatic or degenerative is a common cause of anterior shoulder pain [27], and operative treatment options include tenotomy or tenodesis. Historically biceps tenotomy has been proposed in older patients (over 65 years) or in case of low-demanding activities [20, 28]. This approach had two explanations. From one side the orthopaedic literature highlighted that increasing age, increased the risk of morbidity and mortality in operated patients, particularly that of fracture, wound infection, and venous thromboembolic disease [1, 6–15, 29–32]. From the other, it is well established that tenotomy requires reduced immobilization with reduced adverse side effects and postoperative rehabilitation with decreased risk of postoperative stiffness. However, the drawback of this option is the increased incidence of postoperative cramping and poor cosmesis [18–21, 23, 27]. Conversely, tenodesis has been generally performed in younger and more active subjects. Subpectoral fixation has been initially described [33] to reduce the rate of postoperative pain traditionally associated with arthroscopic techniques [34]. This seems related to the more distal tenodesis site achievable with subpectoral tenodesis [35].

Unfortunately, a variable spectrum of complications has been described including failure or re-rupture of the tendon, hematoma, infection, persistent pain, reaction to a fixation device, nerve injury, cosmetic deformity, and fracture [36, 37]. Humeral fractures have been observed with cortical screws [16, 17] as a consequence of the reduced bone resistance when a hole is drilled [38]. Euler et al. have demonstrated a correlation with laterally eccentric screws [39]. This risk is even higher in old patients with reduced bone mineral density (BMD). According to these evidences, the best treatment option of pathologic LHB is still debated, especially in older patients. This study is comparing the complications rates of LHB subpectoral tenodesis with screw fixation in patients with more than 65 years and less than 65 years. In addition, functional outcomes in the over 65 years cohort were reported. All patients had the same surgical technique performed by the same senior surgeon. In addition, although the analysis of the data was retrospective they were prospectively collected by an independent surgeon. The difference in rates of complications strictly related to LHB tenodesis between the two groups (8.7% in the older group vs 5.4% in the younger group was not statistically significant (*p* = 0.23). This data is however higher than what has been reported by Rios et al. (3%) [40], and Nho et al. (2%) [41]. General complications rate was higher in both groups with the majority of them being persistent pain or complications related to concomitant procedures such as cuff repair (adhesive capsulitis, weakness, failure of the repair. However major adverse effects such as deaths, intraoperative fractures, intraoperative nerve or vessel damage were not observed. The reasonable for his difference might be multifarious and related the accompanied primary surgical rotator cuff repair. Therefore, a greater degree of tendon retraction, the surgical reconstruction of massive cuff tears compared to single tendon ruptures or the general morbidity of the population presented in our department might influence the rate. In addition to complications, clinical outcomes in the older patient group were evaluated. Statistically significant improvement across all outcome measures collected was observed with final ASES being 90.8 (±16.2), SST score 10.6 (±2.2) and CM increased 89.6 (±9.0). In addition, the mean postoperative SANE was 89.9 (±15.3)., indicating a high level of satisfaction with the outcome of the procedure. These data were similar to those reported in previous series on isolated subpectoral tenodesis [42, 43]. In the series by Mazzocca et al. at an average FU of 29 months, mean Constant-Murley score was 90.2, ASES score was 89.2, SST score 10.6, and SANE score 86.9% [43]. Werner et al. reported similar results at an average FU of 3.3 years with a mean Constant-Murley score of 91.8, ASES score of 88.4, SST score of 10.6, and SANE score of 86.8% [42]. Though many surgeons believe that a patient age over 65 is a contraindication to biceps tenodesis, the present study did not show an increased incidence of complications in this specific patient population whereas confirm the satisfactory outcomes previously reported in younger patients.

Several limitations were identified during the course of this study. Firstly, the retrospective design has inherent limitations due to the inability to randomize the sample and manipulate the independent variable. Secondly there are unequal sample sizes between the group of interest and the younger age group. This was due to the distribution of the patients see in our clinic and operated on. Thirdly, the cohort of patients over the age of 65 is relatively small. As there are little reports on outcomes and complications in this patient population, we believe that this is a meaningful contribution to the literature. Finally, as the biceps tenodesis procedure was a concomitant procedure in most cases, it is impossible to distinguish the amount of clinical improvement that can be attributed to the biceps procedure. However, we believe that if patients continued to have pain or limitations due to their biceps tendon, it would be reflected in their postoperative clinical outcomes.

## Conclusion

This study demonstrates that in patients over the age of 65, biceps tenodesis is a successful procedure when performed for biceps tendinopathy and concomitantly with other surgical procedures of the shoulder, and does not result in an increased rate of complications when compared to a group of patients under the age of 65. In addition, the functional outcomes are comparable to those reported in recent studies on younger cohorts.

## Abbreviations

ASES: American shoulder and elbow surgeons
BMD: Bone mineral density
CM: Constant-Murley
FU: Follow up
IRB: Institutional review board
LHB: Long head biceps tendon
SANE: Single assessment numeric evaluation
SST: Simple shoulder test
